# Increased social interaction in *Shank2*-deficient mice following acute social isolation

**DOI:** 10.1186/s13041-023-01025-x

**Published:** 2023-04-15

**Authors:** Ja Eun Choi, Bong-Kiun Kaang

**Affiliations:** School of Biological Sciences, Seoul National University, 1 Gwanangno, Gwanak-Gu, Seoul, 08826 South Korea

**Keywords:** Social isolation, *Shank2*, Social interaction, Sexual dimorphism

## Abstract

Autism spectrum disorder (ASD) is neuropsychiatric disorder with a gender specific risk. Although social impairment in ASD is one of the well characterized phenotypes, loneliness issue resides in patients with ASD and emerging reports show gender distribution in symptoms. Acute social isolation increases the motivation to socially interact in a gender-dependent manner, as only the male mice show increase in sociability following isolation. However, it remains to be explored whether the effects of loneliness in ASD differ between genders. Here, we used *Shank2*-deficient (*Shank2*^−/−^) mice, one of the animal models of ASD, to examine the sociability changes after acute social isolation. While only the male wild-type (WT) mice display increased sociability following 24-h isolation, both sexes of *Shank2*^−/−^ mice show an increase in social interaction following isolation. These observations provide evidence that animal models of ASD have the sensitivity to acute social isolation and further show the motivation to socially interact.

**Supplementary Information:**

The online version contains supplementary material available at 10.1186/s13041-023-01025-x.

## Micro report main text

Autism spectrum disorder (ASD) is a neurodevelopmental disorder with a strong genetic effect. One of the core characteristics of ASD is social deficit [1, 2], in which the phenotype is well reproduced in various animal models of ASD [3, 4]. Although accumulating reports reveal the link between ASD and genetic mutations to help understand the mechanism of ASD [3, 5], the number of cases with gender distribution still remains small. In fact, several literatures have reported the existence of gender-specific risk in ASD patients [6, 7], supporting the necessity of research. Given the scope of this sexual dimorphic symptoms, understanding the biological variations by gender in ASD and further applying to treatments have been challenging.

As maintaining social bond is an important factor in socially innate animals, social isolation and loneliness are complicated issues in patients with ASD [8, 9]. Acute (24 h) social isolation increases the motivation to socially interact [10]. Recently, it is reported that only the male mice exhibit the sociability increase while the female mice show an anxiety increase following acute isolation, providing gender-specific phenotypes [11].

SHANK2 is one of the scaffolding proteins in the excitatory neurons which shows postsynaptic localization. The association of SHANK2 with ASD, especially with social deficits, has been well reported by many groups [3, 5, 12, 13]. Here, we used *Shank2*^*−/−*^ mice to further investigate whether there is a gender difference in the effect of loneliness on ASD.

**Fig. 1:**
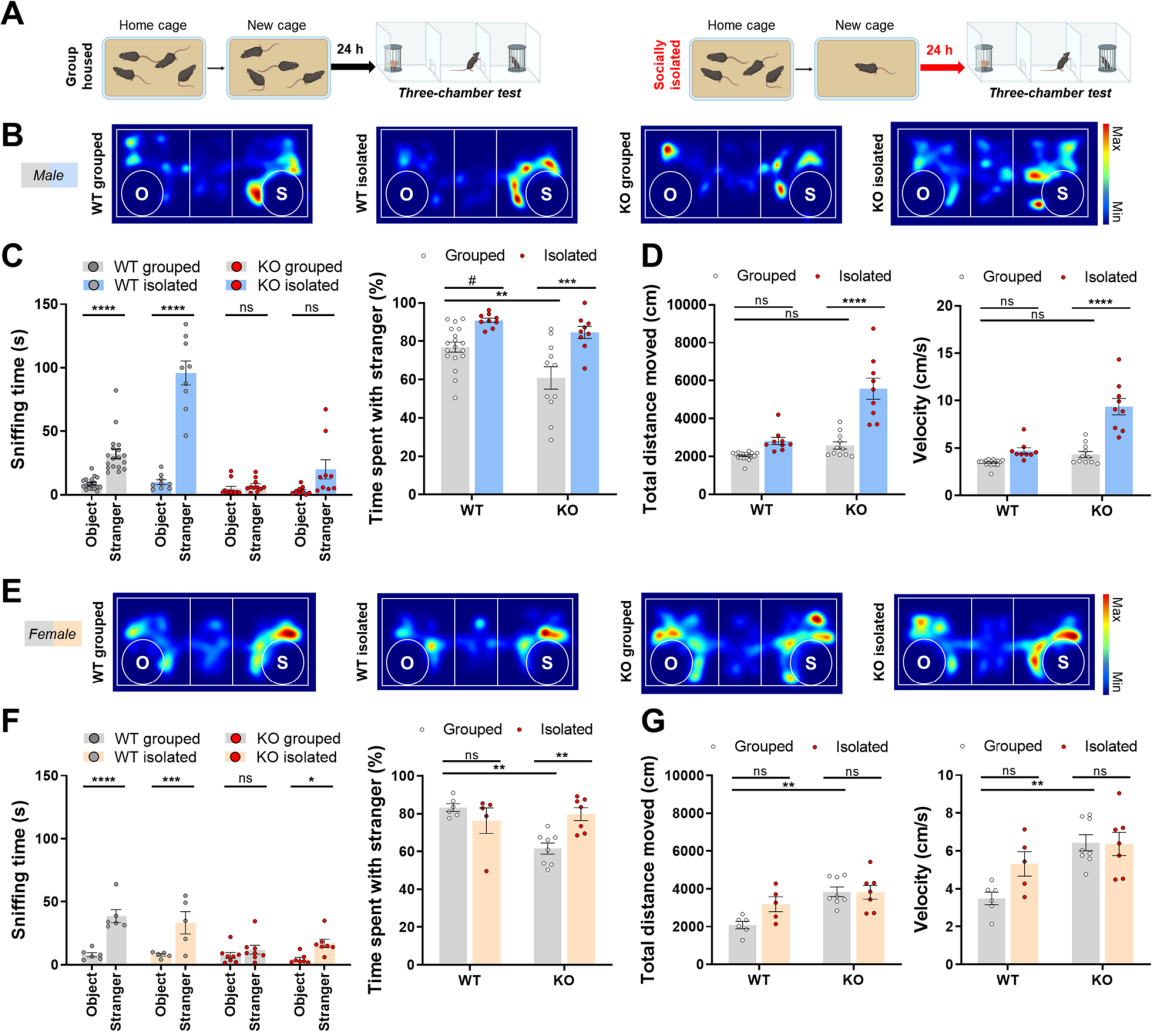
Behavioral changes following acute social isolation in *Shank2*^*−/−*^ mice. **A** Experimental scheme of three-chamber test following group-housing and social isolation. **B** Heat maps of WT grouped, WT isolated, KO grouped and KO isolated male mice. **C** Isolation increases the sociability level both in WT and KO mice (Sniffing time; WT grouped, p**** < 0.0001; WT isolated, p**** < 0.0001; KO grouped, *p* > 0.9999; KO isolated, *p* = 0.0918; time spent with stranger; WT grouped vs. WT isolated, *p*^*#*^ = 0.0519; KO grouped vs. KO isolated, p*** = 0.0007; WT grouped vs. KO grouped, p** = 0.0094). **D** Total distance moved and velocity are increased in KO isolated male mice (Total distance moved; WT grouped vs. WT isolated, *p* = 0.1487; KO grouped vs. KO isolated, p**** < 0.0001; WT grouped vs. KO grouped, *p* = 0.5388; velocity; WT grouped vs. WT isolated, *p* = 0.1371; KO grouped vs. KO isolated, p**** < 0.0001; WT grouped vs. KO grouped, *p* = 0.5224). **E** Heat maps of four types of groups in female mice. **F** The sociability level is increased in KO mice following social isolation (Sniffing time; WT grouped, p**** < 0.0001; WT isolated, p*** = 0.0001; KO grouped, *p* > 0.9999; KO isolated, p* = 0.0381; time spent with stranger; WT grouped vs. WT isolated, *p* > 0.9999; KO grouped vs. KO isolated, p** = 0.0080; WT grouped vs. KO grouped, p** = 0.0023). **G** The mobility level is not affected by social isolation (Total distance moved; WT grouped vs. WT isolated, *p* = 0.1822; KO grouped vs. KO isolated, *p* > 0.9999; WT grouped vs. KO grouped, p** = 0.0025; velocity; WT grouped vs. WT isolated, *p* = 0.1838; KO grouped vs. KO isolated, *p* > 0.9999; WT grouped vs. KO grouped, p** = 0.0024). Additional statistical information is in Additional file 2

We performed three-chamber test following 24 h of group-housing or social isolation [10, 11] to examine the behavioral effect of acute social isolation in *Shank2*^*−/−*^ mice. As acute social isolation leads sexual dimorphic phenotypes [11], we further wondered whether sex-dependent observations are detected in *Shank2*^*−/−*^ mice (Fig. 1A). In male mice, higher sociability level in the isolated WT mice compared to grouped WT mice was observed (Fig. 1B, C), reproducing the previous report [11]. Grouped *Shank2*^*−/−*^ mice showed social impairments, but after they underwent 24 h of social isolation, *Shank2*^*−/−*^ mice showed higher preference to the stranger mice than the object (Fig. 1C). These observations indicate that transferring to a new cage 1 day before three-chamber test did not interfere with the expression of social deficits in *Shank2*^*−/−*^ mice and the sensitivity to social isolation was intact even with the SHANK2 deficiency. The acute social isolation gave rise to increase in *Shank2*^*−/−*^ mice in the total distance moved and the velocity (Fig. 1D).

In the female mice, we performed the same behavioral tasks to figure out the sexual dimorphic phenotypes following acute social isolation. Grouped WT and isolated WT mice showed comparable sociability level, matching with our previous report [11], and the expression of social impairments in *Shank2*^*−/−*^ mice was detected regardless of sex (Fig. 1E, F). However, unlike the tolerance to acute social isolation in WT female mice, the isolated *Shank2*^*−/−*^ female mice spent greater time with sex-matched stranger mice (Fig. 1F). The total distance moved and the velocity were only increased between grouped WT and grouped *Shank2*^*−/−*^ female mice, indicating the acute social isolation did not change the mobility during three-chamber test (Fig. 1G).

The behavioral phenotypes observed in ASD mainly focus on the social deficits as individuals with ASD find social stimuli less rewarding. However, the loneliness issue in patients diagnosed with ASD is also an important point related with their low interest in social interaction. Our observation of increased sociability level in *Shank2*^*−/−*^ mice after isolation highlights a potential relationship between social deficits, motivation, and loneliness in ASD. Of note, even though the percentage of time spent with stranger have significantly increased in both sexes of *Shank2*^*−/−*^ mice following acute social isolation, it did not fully reach to the level of WT mice. It implies that the social difficulties in *Shank2*^*−/−*^ mice still reside despite the motivation to socially interact was expressed. Thus, our observation should be carefully interpreted which further gives rise to the need for additional experiments, such as other behavioral tasks with circuit level manipulation.

There are two discussion points regarding the gender-dependent phenotypes observed in *Shank2*^*−/−*^ mice after isolation. First, while isolation led to increased social interaction in male mice, both sexes of *Shank2*^*−/−*^ mice showed an increase in sociability. Second, only male *Shank2*^*−/−*^ mice showed a significant increase in locomotion after isolation. Thus, these observations suggest a disruption of behaviors and related brain regions in ASD but further investigations are needed. The behavioral deficits in ASD reflects the heterogeneous characteristics of ASD as various brain regions and cell types contribute to social impairments [14, 15]. Given that individuals with ASD experience higher levels of loneliness than non-autistic individuals, we posited that the DRN, which has been implicated as a brain region involved in loneliness, may also play a role in loneliness in ASD. Acute social isolation gives rise to potentiating excitatory inputs onto the dorsal raphe nucleus (DRN) dopaminergic neurons [10] and strengthening outputs of DRN dopaminergic neurons co-releasing glutamate onto the nucleus accumbens [11]. Although complex interactions may exist due to SHANK2 deficiency, we speculate that neuronal input or/and output circuits related with dopaminergic populations of DRN may be related to the observed phenotypes in *Shank2*^*−/−*^ mice.

## Supplementary Information

**Additional file 1.** Materials and methods.

**Additional file 2.** Statistical analysis.

## Data Availability

The datasets used and/or analyzed during the current study are available from the corresponding author on reasonable request. The materials and methods are presented in Additional file 1.

## Abbreviations

ASD: Autism spectrum disorder
DRN: Dorsal raphe nucleus
WT: Wild-type
