# Supplementary material for: Increased social interaction in Shank2-deficient mice following acute social isolation
Source: Mol Brain. 2023 Apr 15;16:35. doi: 10.1186/s13041-023-01025-x (PMC10105924; doi:10.1186/s13041-023-01025-x)
Supplement: Supplementary file 1 — Additional file 1. Materials and methods. [file 13041_2023_1025_MOESM1_ESM.docx]

**Additional file 1.**

**Materials and methods**

**Animals**

Homozygous Shank2-deficient (*Shank2^-/-^*) mice were generated by crossing heterozygous Shank2 (*Shank2^+/-^*) mice with *Shank2^+/-^* mice and were maintained in a C57BL/6 background (*1*). Eight to sixteen week-old wild type (*Shank2^+/+^*) and *Shank2^-/-^* mice were used for behavior tasks. Animals were housed with food and water *ad libitum* on a 12 h light-dark cycle. All experimental procedures were approved by the Institutional Animal Care and Use Committee (IACUC) of Seoul National University. For male mice, WT grouped (n = 18), WT isolated (n = 9), KO grouped (n = 11) and KO isolated (n = 9) were used. For female mice, WT grouped (n = 6), WT isolated (n = 5), KO grouped (n = 8) and KO isolated (n = 7) were used.

**Behavior test**

Three-chamber test was performed as described previously (*2*). During the 10 minutes of habituation session, test mice were allowed to freely move in the three-chamber apparatus. Then, an unfamiliar sex-matched juvenile mouse (stranger) was placed under one wired cup and an object was placed under the other wired cup. During the 10 minutes of test session, the test mice were recorded for the preference to each wired cup. The time of sniffing to each cup (stranger and object) was analyzed. The percentage of time spent with stranger was divided by the total time of sniffing both stranger and object. Ethovision software was used for the heat maps, velocity and total distance moved.

**References**

1. H. Won *et al.*, Autistic-like social behaviour in Shank2-mutant mice improved by restoring NMDA receptor function. *Nature* **486**, 261-265 (2012).

2. J. E. Choi *et al.*, Synaptic ensembles between raphe and D(1)R-containing accumbens shell neurons underlie postisolation sociability in males. *Sci Adv* **8**, eabo7527 (2022).
