# Supplementary material for: Increased social interaction in Shank2-deficient mice following acute social isolation
Source: Mol Brain. 2023 Apr 15;16:35. doi: 10.1186/s13041-023-01025-x (PMC10105924; doi:10.1186/s13041-023-01025-x)

**Additional file 2**

**Statistical analysis**

Statistical analyses were performed using Prism 8 (Graphpad). Three-way ANOVA was used in analyzing the sniffing time and two-way ANOVA, Bonferroni's multiple comparisons test was used in the rest of the data.


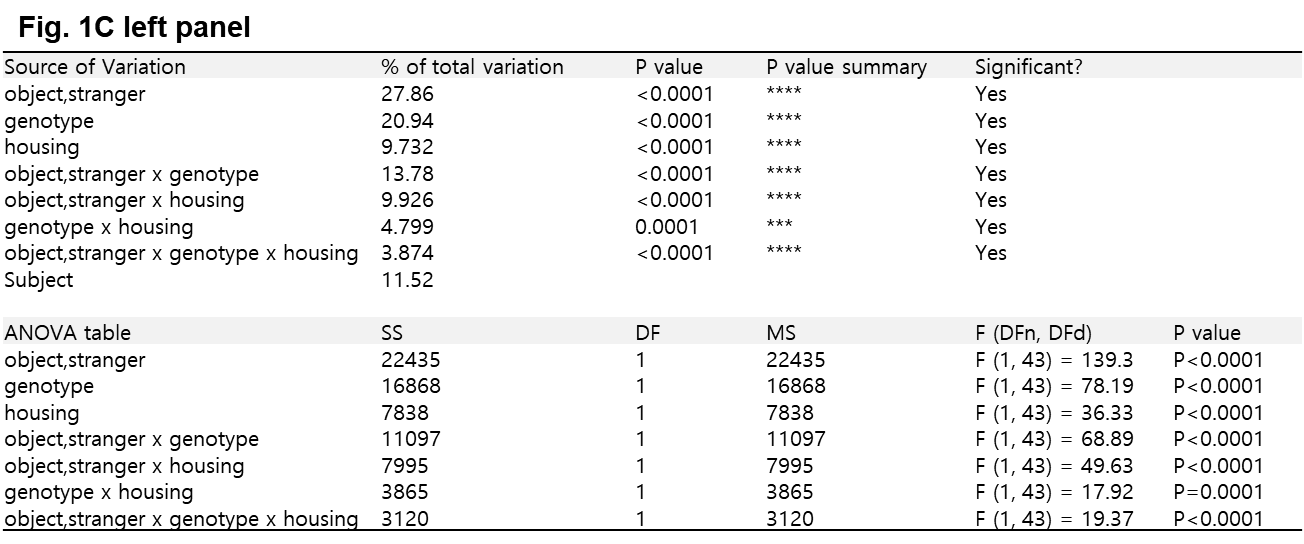

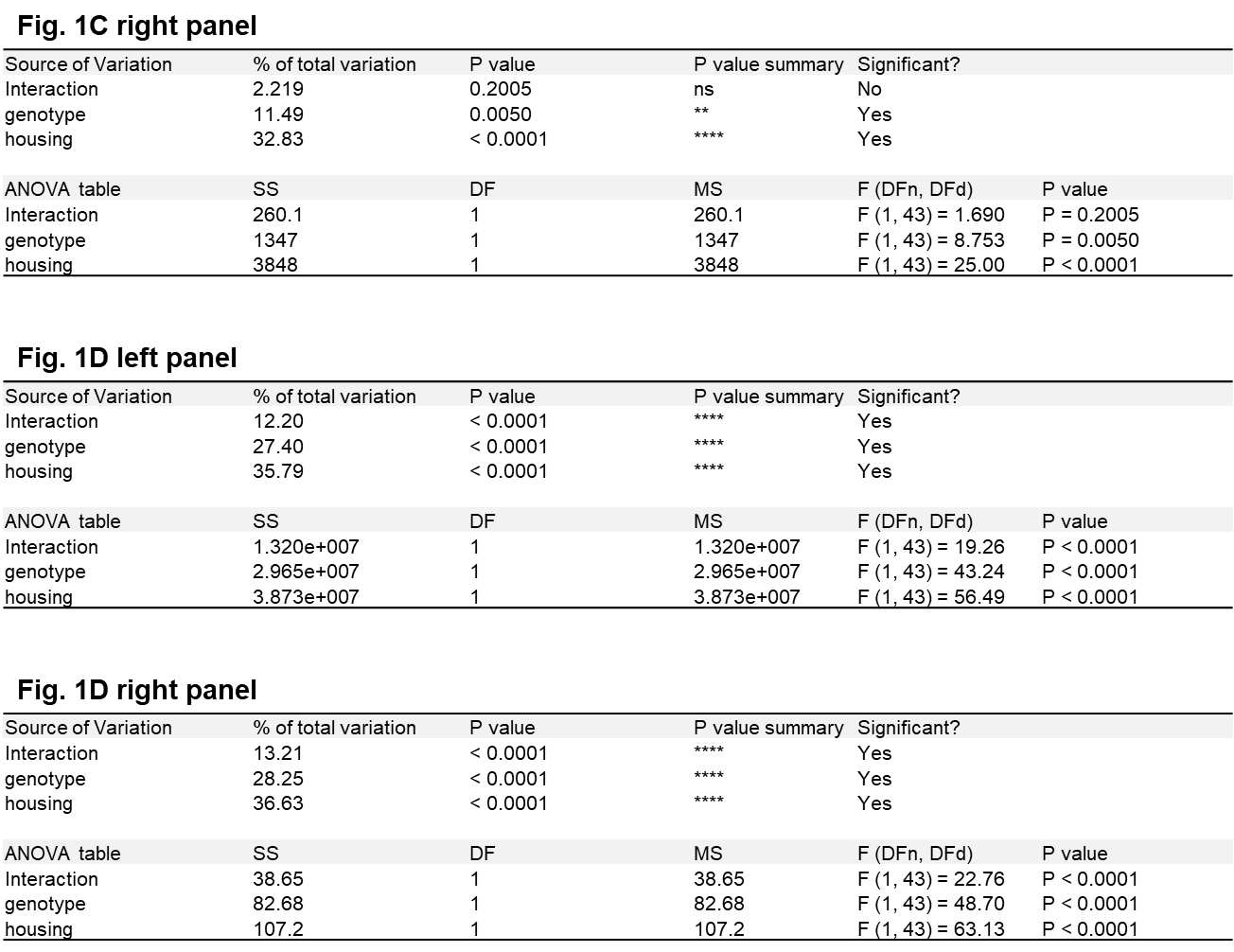


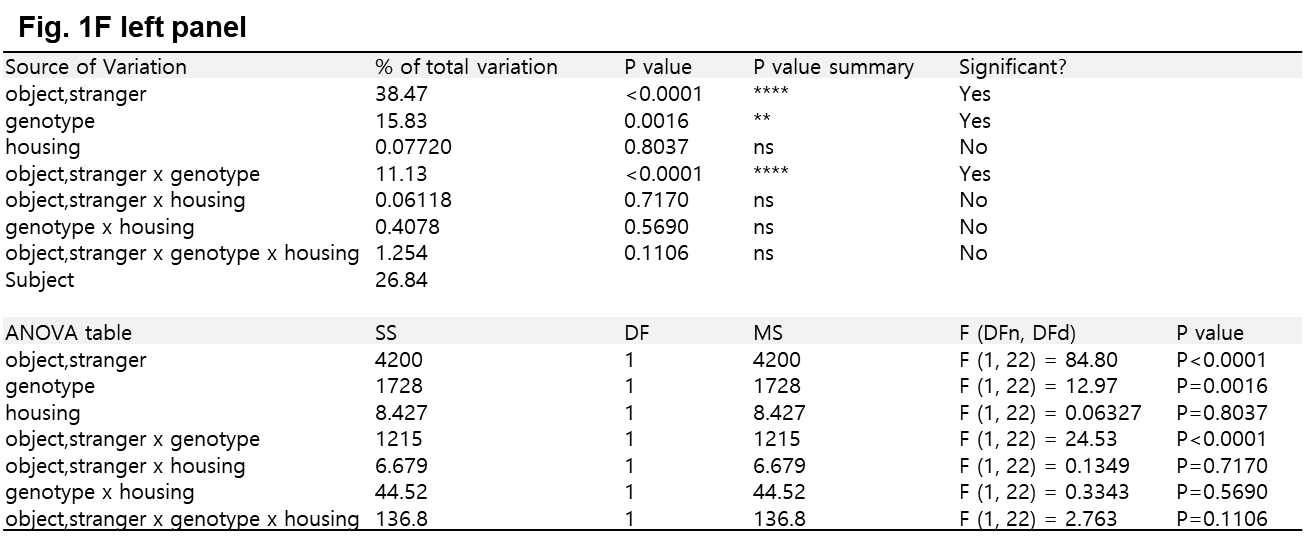

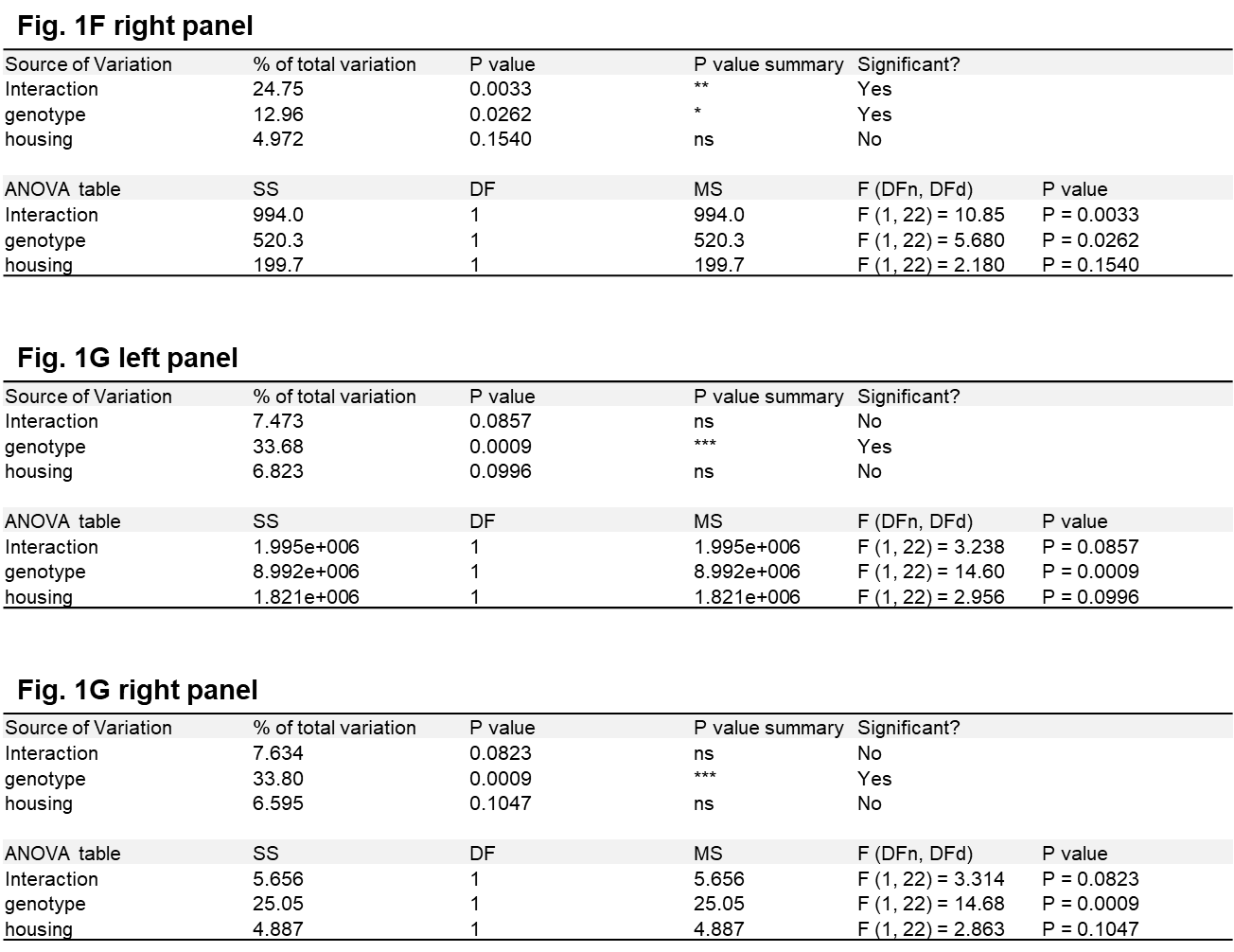


**The raw data used in this paper.**


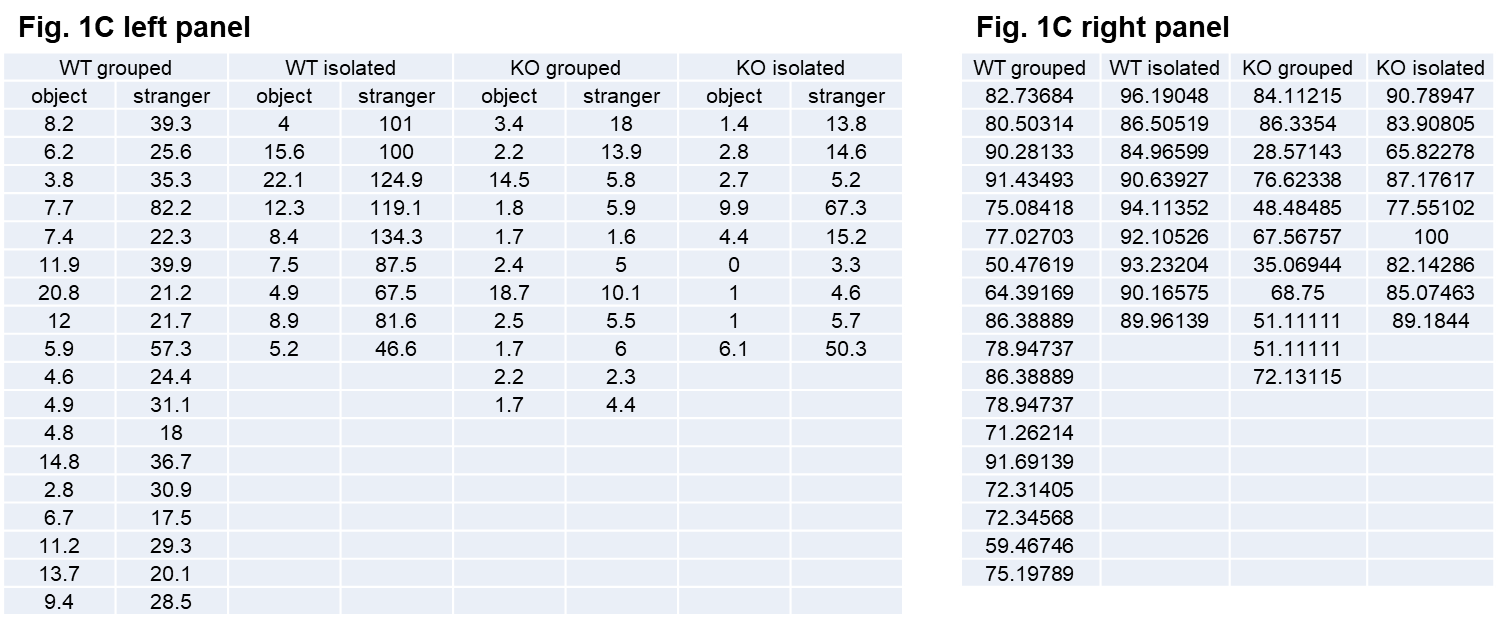

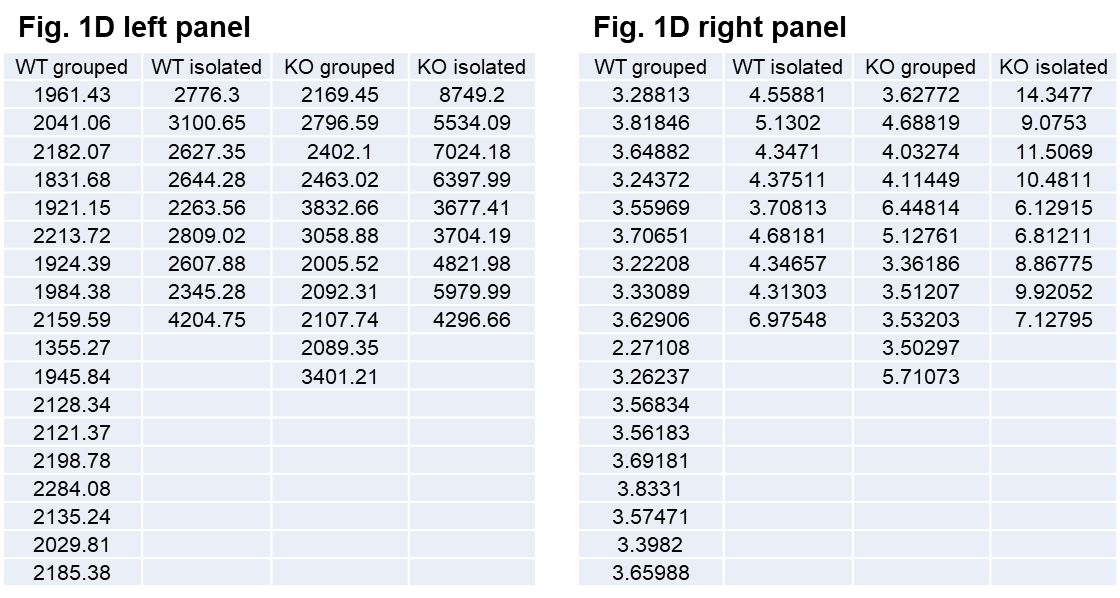

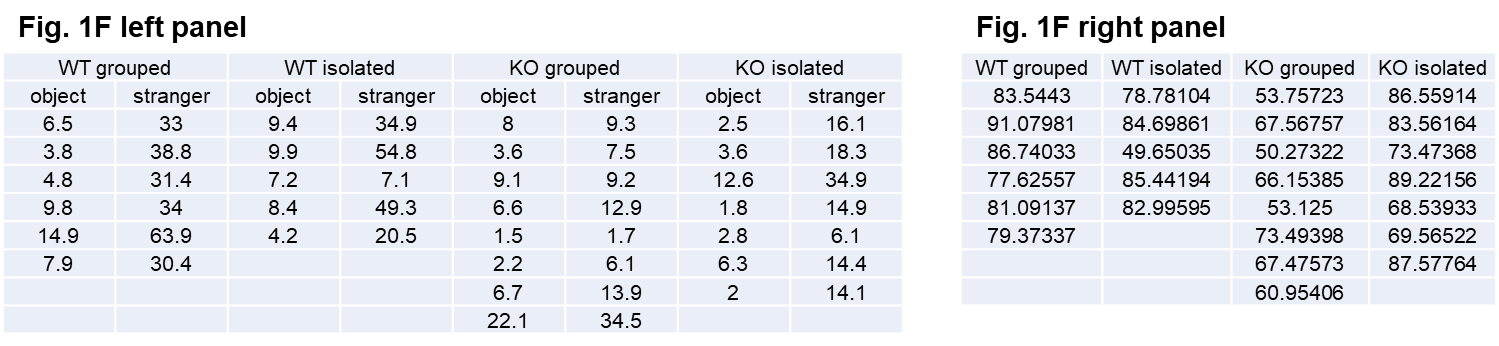

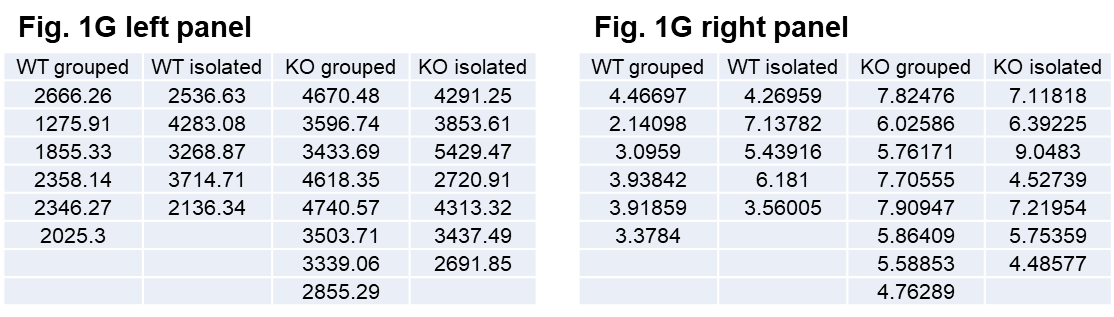

Supplement: Supplementary file 2 — Additional file 2. Statistical analysis. [file 13041_2023_1025_MOESM2_ESM.docx]
